# Distribution of O-Acetylated Sialic Acids among Target Host Tissues for Influenza Virus

**DOI:** 10.1128/mSphere.00379-16

**Published:** 2017-09-06

**Authors:** Brian R. Wasik, Karen N. Barnard, Robert J. Ossiboff, Zahra Khedri, Kurtis H. Feng, Hai Yu, Xi Chen, Daniel R. Perez, Ajit Varki, Colin R. Parrish

**Affiliations:** a Department of Microbiology and Immunology, Baker Institute for Animal Health, College of Veterinary Medicine, Cornell University, Ithaca, New York, USA; b Department of Population Medicine and Diagnostic Sciences, Animal Health Diagnostic Center, College of Veterinary Medicine, Cornell University, Ithaca, New York, USA; c Glycobiology Research and Training Center, University of California, San Diego, La Jolla, California, USA; d Department of Chemistry, University of California, Davis, Davis, California, USA; e Department of Population Health, Poultry Diagnostic and Research Center, College of Veterinary Medicine, University of Georgia, Athens, Georgia, USA; Boston University School of Medicine

**Keywords:** host range, influenza, receptor-ligand interaction, respiratory viruses, sialic acid, virus-host interactions

## Abstract

Sialic acids (Sias) are important glycans displayed on the cells and tissues of many different animals and are frequent targets for binding and modification by pathogens, including influenza viruses. Influenza virus hemagglutinins bind Sias during the infection of their normal hosts, while the encoded neuraminidases and/or esterases remove or modify the Sia to allow virion release or to prevent rebinding. Sias naturally occur in a variety of modified forms, and modified Sias can alter influenza virus host tropisms through their altered interactions with the viral glycoproteins. However, the distribution of modified Sia forms and their effects on pathogen-host interactions are still poorly understood. Here we used probes developed from viral Sia-binding proteins to detect O-acetylated (4-O-acetyl, 9-O-acetyl, and 7,9-O-acetyl) Sias displayed on the tissues of some natural or experimental hosts for influenza viruses. These modified Sias showed highly variable displays between the hosts and tissues examined. The 9-O-acetyl (and 7,9-) modified Sia forms were found on cells and tissues of many hosts, including mice, humans, ferrets, guinea pigs, pigs, horses, dogs, as well as in those of ducks and embryonated chicken egg tissues and membranes, although in variable amounts. The 4-O-acetyl Sias were found in the respiratory tissues of fewer animals, being primarily displayed in the horse and guinea pig, but were not detected in humans or pigs. The results suggest that these Sia variants may influence virus tropisms by altering and selecting their cell interactions.

**IMPORTANCE** Sialic acids (Sias) are key glycans that control or modulate many normal cell and tissue functions while also interacting with a variety of pathogens, including many different viruses. Sias are naturally displayed in a variety of different forms, with modifications at several positions that can alter their functional interactions with pathogens. In addition, Sias are often modified or removed by enzymes such as host or pathogen esterases or sialidases (neuraminidases), and Sia modifications can alter those enzymatic activities to impact pathogen infections. Sia chemical diversity in different hosts and tissues likely alters the pathogen-host interactions and influences the outcome of infection. Here we explored the display of 4-O-acetyl, 9-O-acetyl, and 7,9-O-acetyl modified Sia forms in some target tissues for influenza virus infection in mice, humans, birds, guinea pigs, ferrets, swine, horses, and dogs, which encompass many natural and laboratory hosts of those viruses.

## Abstract (precis)

Sialic acids (Sias) are key glycans that control or modulate many normal cell and tissue functions while also interacting with a variety of pathogens, including many different viruses. Sias are naturally displayed in a variety of different forms, with modifications at several positions that can alter their functional interactions with pathogens. In addition, Sias are often modified or removed by enzymes such as host or pathogen esterases or sialidases (neuraminidases), and Sia modifications can alter those enzymatic activities to impact pathogen infections. Sia chemical diversity in different hosts and tissues likely alters the pathogen-host interactions and influences the outcome of infection. Here we explored the display of 4-O-acetyl, 9-O-acetyl, and 7,9-O-acetyl modified Sia forms in some target tissues for influenza virus infection in mice, humans, birds, guinea pigs, ferrets, swine, horses, and dogs, which encompass many natural and laboratory hosts of those viruses.

## INTRODUCTION

All cells are decorated on their surfaces with a complex array of oligosaccharides (glycans) that are attached to membrane glycoproteins and glycolipids or which make up the different components of soluble extracellular materials such as mucus and which are involved in the initial interactions of viruses or other pathogens with cells or mucosal surfaces (1). The chemical diversity of these glycans contributes to their complex functional roles in controlling cell-cell, cell-pathogen, and cell-environment interactions. Linear and branched glycoconjugates of vertebrate cells are often terminated in sialic acids (Sias). Sias have critical roles in maintaining a variety of cell functions due to their abundance as exposed terminal sugars, where they are involved in highly specific and regulated cellular lectin interactions (2, 3). They have also been exploited by many different pathogens as well as by nonpathogenic microbes for host recognition and attachment, including playing a role as receptors for important disease-causing viruses, bacteria, and parasites (4, 5).

Sias are nine-carbon α-keto acids that may be produced and displayed in multiple modified forms that differ in expression between organisms, tissues, and even individual cells (1, 6, 7). In one series of modifications, the most common form, *N*-acetylneuraminic acid (Neu5Ac), may be modified with acetyl groups (O-linked) at carbons -4, -7, -8, and/or -9 (8, 9). The presence and levels of O-acetylation likely result from combined functions of sialic acid O-acetyl transferases (SOAT) and esterases (SOAE), some of which have been identified and functionally demonstrated, while others are not yet characterized (10–12). A variety of studies have contributed to our understanding of the relative abundances and distributions of O-acetylated Sias in various eukaryotic systems, as reviewed by Mandal et al. (13). However, the recent development of recombinant sialoglycan-recognizing probes (SGRPs) recognizing different O-acetylated Sia forms allows their display at the cellular and tissue levels to be analyzed in more detail (14).

Sia-lectin interactions are critical within organisms, and O-acetyl modifications modulate a variety of intrinsic host functions. Our understanding is clearest for the 9-O-acetyl-modified Sias, which have been detected in human and some other animal tissues with influenza C virus hemagglutinin-esterase fusion (HEF) proteins (8) and subsequently with porcine *Torovirus* HE (14). Those studies showed that 9-O-acetyl (9-O-Ac) Sias were common in many human tissues and on many cell lines of human origins, as well as being expressed by other animals and displayed within their tissues with highly variable patterns. Removal of 9-O-Ac Sias by expression of the influenza C 9-O-acetylesterase in transgenic mice resulted in developmental arrest of embryos around the 2-cell stage (15). Regulated 9-O-acetylation is also widely involved in various immunological pathways. For example, modification of the ganglioside GD3 (CD60) of proliferating T cells affects apoptotic pathways and their differentiation states (16), while Sia 9-O-acetylation on B-cell surface glycans blocks recognition by Sia-binding immunoglobulin-type lectin Siglec-2 (CD22), inhibiting receptor signaling and B-cell activation (17). Given their involvement in and regulation of cell signaling and proliferation processes, it is not surprising that abnormally displayed O-acetylated Sias are often oncogenic markers (18–21). Further additions of O-acetyl groups to the 7 and 8 positions give rise to di- and tri-O-acetylated Sias, respectively.

The display and functions of modified 4-O-Ac Sias in intrinsic cell processes are poorly understood. They have been previously identified using high-performance liquid chromatography (HPLC) analyses in certain tissues, blood proteins, and erythrocytes and within the cells of vertebrates of varied lineages, including horses and donkeys, guinea pigs, mice, and monotremes, and in many lineages of bony fish (14, 22–26). They appear to be distributed in fewer species than the O-acetyl modifications on the Sia glycerol side chain.

Influenza virus particles are decorated with the major glycoproteins hemagglutinin (HA) (displayed as trimers) and neuraminidase (NA) (displayed as tetramers) (27). Sias act as functional receptors for influenza virus infection as they are recognized and bound by the HA and are cleaved from cell glycans (and viral glycoproteins) by the sialidase function of NA (28–30). The specific chemistry and linkages of the cell Sias have long been known to influence HA and NA specificity and, ultimately, influenza virus infectivity. Influenza A virus (IAV) and influenza B virus (IBV) HAs from many hosts show strong preferences for binding and infection using Neu5Ac as a receptor, while modified Sias, including Neu5Gc and O-acetyl forms, may reduce or alter infection by those viruses (31). The Sia sugar linkage to the penultimate residue (α2-3 versus α2-6) affects HA Sia binding affinity and also contributes to host tropism differences between avian and human influenza virus strains (32, 33). O-acetyl modified Sias have been reported to affect influenza virus infections by altering the activities of HA and NA. One reported effect of O-acetylation is a general reduction in susceptibility to cleavage by sialidases, including influenza virus NA (34). A number of inhibitors of influenza viruses were identified historically, including the γ-inhibitors, which are defined as sugar molecules that interact with influenza viruses to inhibit infection by acting as “decoy” receptors (35–38). The 9-O-Ac Sias were also reported to reduce the sialidase activity of NA by almost 3-fold (39). Strong inhibition of infection and cell-to-cell spread of some human IAVs by horse and guinea pig sera was associated with the presence of high levels of 4-O-Ac Sias on plasma glycoproteins, including α-2-macroglobulin (35, 37, 40–43).

In other cases, the O-acetyl-modified Sias are required for cell infection by viruses. Both influenza C viruses and influenza D viruses encode a HEF glycoprotein that binds 9-O-Ac Sias as a functional receptor and that also contains a 9-O-Ac-specific esterase that promotes viral release from that Sia form (44, 45). The orthomyxovirus salmon anemia virus and the coronavirus mouse hepatitis virus-S strain each use 4-O-Ac Sia as a specific receptor for cell binding and infection and also express esterases that can remove that modification (46, 47).

The modified Sia may also alter the cell and tissue interactions with other viruses, as well as modifying infections of various bacteria and parasites. For example, reovirus type 3 infection and replication were inhibited by the presence of 9-O-Ac Sia (48), as was *Plasmodium falciparum* binding to and invasion of erythrocytes (49). Many bacteria—both pathogens and commensals—bind to Sia and often express sialidases (neuramindases) that cleave it from glycans, and those activities are likely susceptible to the effects of modifications of the Sia substrate (50–53). In the bacterial oral pathogen *Tannerella forsythia*, an acetylesterase (NanS) is encoded within the Sia operon to remove human 9-O-Ac modifications and improve NanH sialidase function (54). Understanding the differences in the display and cell or tissue distributions of these modified Sia forms between various animals would therefore be of general interest for understanding how they may alter animal susceptibility to pathogens. Identifying their patterns of expression in tissues of hosts for influenza virus would also help to clarify their roles in controlling HA and NA specificities and activities and hence their effects on tropisms and host ranges of those viruses (55).

Here we used previously described recombinant soluble viral SGRPs or “virolectins” (14) with specificities for different O-acetylated Sias, as well as esterases active against the same modifications, to survey the diversity of these modified forms on the cells and tissues that are targets for infection by influenza virus and other viruses in their natural hosts, as well as in some animal models.

## RESULTS

### SGRPs identifying specific O-acetylated Sia moieties.

We used the recombinant soluble HE proteins from three nidoviruses as SGRPs to survey for the display of O-acetylated modified Sias in the respiratory tract tissues that are the natural sites of influenza virus infection in mammals, as well as in the gastrointestinal tract tissues in Pekin duck (*Anas platyrhynchos domesticus*) and the tissues of embryonated chicken eggs. These SGRPs have been previously characterized with defined Sia binding and esterase activities (14). Mouse hepatitis virus S strain (MHV-S) HE recognizes 4-O-Ac Sia, bovine coronavirus Mebus strain (BCoV-Mebus) HE primarily recognizes 7,9-O-Ac Sia, and porcine *Torovirus* P4 strain (PToV-P4) HE recognizes 9-O-Ac Sia. Hosts examined included those that are naturally infected and which sustain transmission of influenza viruses in nature (humans, Pekin ducks, swine, horses and dogs) and those used as experimental models for human influenza virus infection and transmission (mice, ferrets, and guinea pigs).

**FIG 1:**
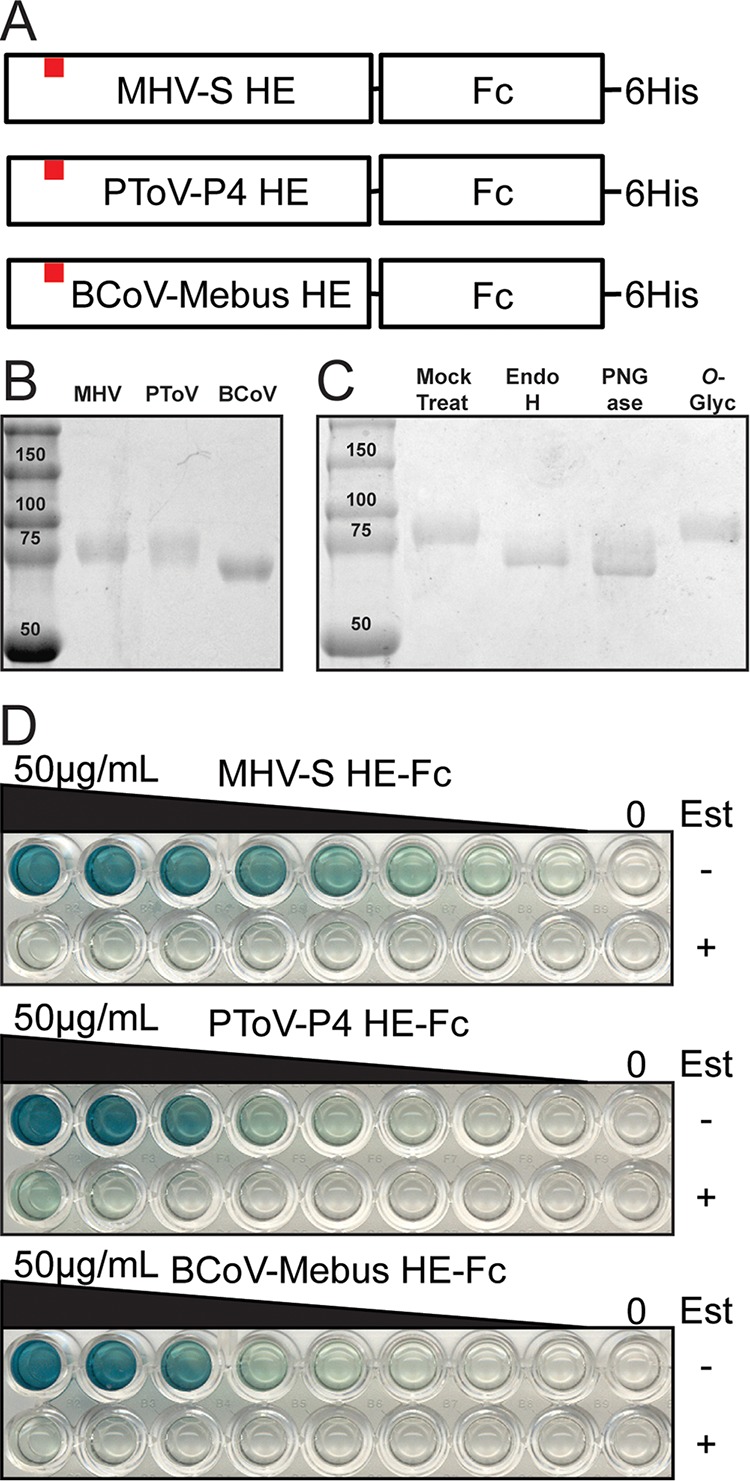
Expression and characterization of SGRPs. (A) Cartoon schematic of SGRPs. Nidovirus HE ectodomains were expressed with a C-terminal tag of the human IgG Fc domain and a 6-His sequence. The esterase active site Ser (denoted by red squares) was mutated to Ala to generate binding SGRPs. (B) Expressed and purified SGRPs resolved on SDS-PAGE. (C) MHV-S HE-Fc treated with various glycosidases and resolved on SDS-PAGE shows presence of N-linked (but not O-linked) glycosylation. (D) Solid-phase lectin binding assays of SGRPs. Plates were coated with horse serum (MHV-S) or bovine submaxillary mucin (PToV-P4, BCoV-Mebus) and probed with 2-fold dilutions of SGRPs (starting at 50 μg/ml). Parallel rows were treated during blocking with active-esterase forms of the proteins (Est+).

The HE proteins are fused to the Fc region of human IgG1 and to a hexahistidine (His_6_) sequence, allowing ready expression, purification, and detection (Fig. 1A). Proteins were expressed from insect cells using a baculovirus vector and purified using protein G columns, resulting in isolation of a single protein (Fig. 1B). Those proteins contained N-linked (but not O-linked) glycans, as shown by treatment with enzymes specific for different glycosylation forms (Fig. 1C). As the proteins were produced in insect cells, they would display high-mannose N-linked glycans and would not themselves be sialylated. Testing the specificities of the SGRPs in a solid-phase lectin binding assay (spLBA) with either horse serum proteins (displaying 4-O-Ac Sias) or bovine submaxilliary gland mucin (7,9-O-Ac Sias and 9-O-Ac Sias) showed the expected binding specificities (Fig. 1D), and those were also confirmed by pretreating the Sia substrates with the esterase-active form of the proteins before conducting the assay, removing the binding substrate (Fig. 1D). The use of solutions at pH 6.5 during esterase treatments prevents O-acetyl migration on the Sia glycerol side chain (56, 57), and esterase controls performed alternatively at pH 7.5 confirmed previous findings of BCoV-Mebus HE-Fc (but not PToV-P4 HE-Fc) binding to 7,9-O-Ac Sias present in bovine mucin (58). We observed no binding of the SGRPs to nonmodified Sia substrates (bovine fetuin, various animal serums) or nonsialylated glycoprotein substrates (results not shown). Goat serum was used as a block of nonspecific binding of the anti-Fc secondary antibody in all subsequent tissue staining experiments. The Sia of that serum did not react with the probes on tissue slides or in testing performed by spLBA, suggesting that the serum does not contain O-acetylated Sias (results not shown).

**FIG 2:**
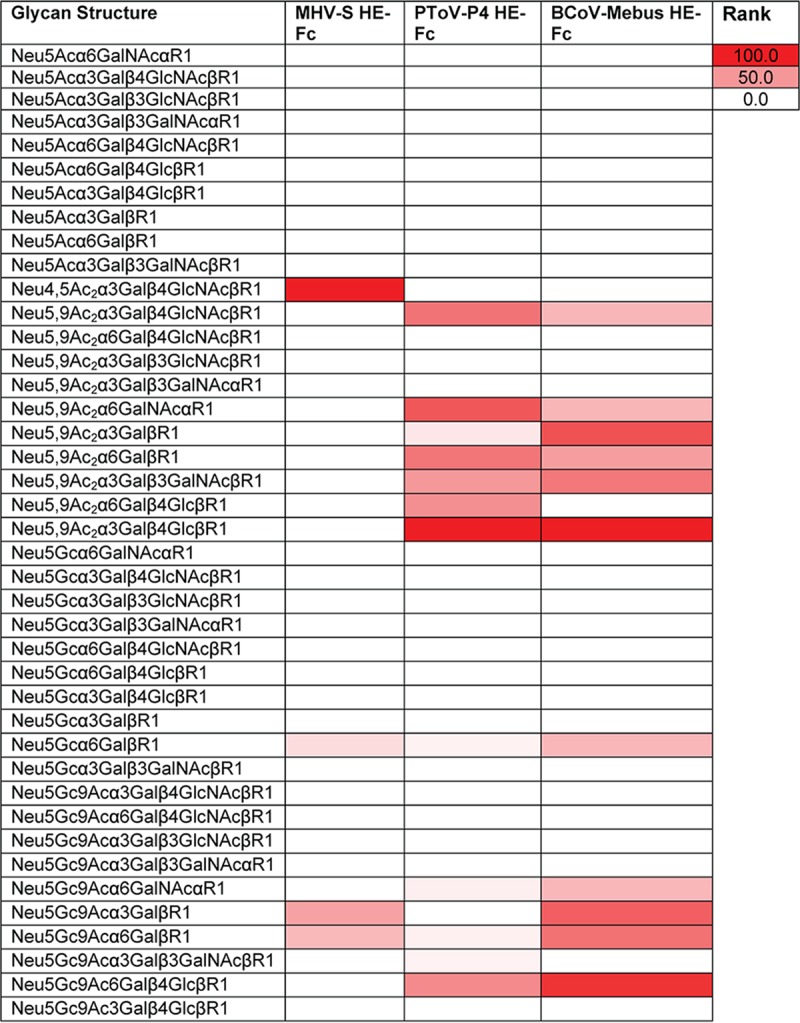
Glycan microarray screening of MHV-S HE-Fc, PToV-P4 HE-Fc, and BCoV-Mebus HE-Fc. The binding is presented in heat map form. The heat map was generated according to a method previously described (87). Binding was ranked and calculated as follows, where RFU represents relative fluorescence units: (glycan RFU/maximum glycan RFU) * 100. Binding data represent results determined at 40 µg/ml (*n* = 4, standard deviations [SD]).

The binding specificity of the probes was tested on a glycan microarray containing Neu4,5Ac_2_ (*n* = 1), Neu5,9Ac_2_ (*n* = 20), and Neu5Gc9Ac (*n* = 1), along with their equivalent but nonacetylated glycan forms (Fig. 2). Binding was ranked as relative fluorescence units (RFU) (*n* = 4, standard deviations [SD]). MHV-S HE-Fc bound very specifically to Neu4,5Ac_2_α3Galβ4GlcNAcβR1 (R1=propyl azide) as the only Neu4,5Ac_2_ glycan on the current array. Both PToV-P4 HE-Fc and BCoV-Mebus HE-Fc bind specifically to 9-O-Ac sialic acids. While PToV-P4 HE-Fc binds only to Neu5,9Ac_2_, BCoV-Mebus HE-Fc binds to both Neu5,9Ac_2_ and Neu5Gc9Ac. Note that 9-O-acetylation is partially lost on many spots because of instability. Thus, results on those spots are meaningful only if binding is much better than that to the corresponding non-O-acetylated glycan spot. Glycan microarray results correlated with our spLBA observations and largely confirmed the Sia moiety specificity of the SGRPs. Glycan microarray results obtained with our SGRPs consistently overlapped previous results reported for these nidovirus HEs (14).

### The distribution of O-acetyl Sias in tissue culture cells.

**FIG 3:**
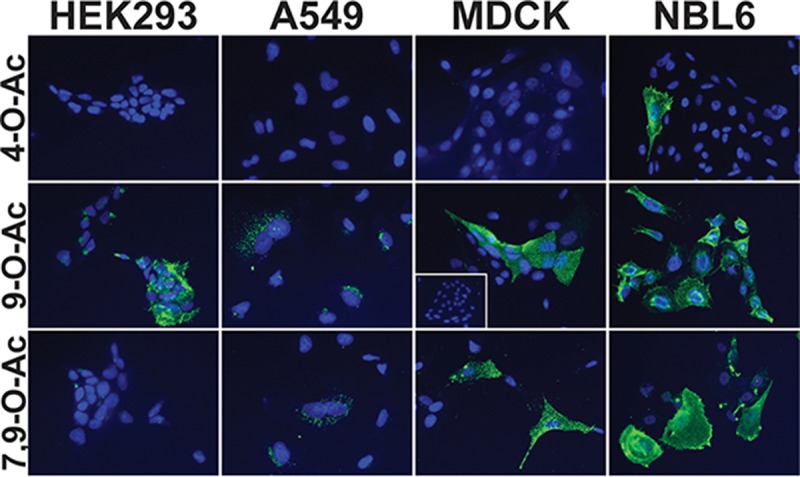
Presence of O-acetyl modified Sias on a selection of cell lines from different hosts commonly used for the growth and assay of influenza viruses. Cell lines of human (HEK-293, A549), canine (MDCK), and equine (NBL-6) origin were screened with the SGRPs for the display of O-acetylated Sias. The display of 4-O-Ac Sias was isolated to horse origin NBL-6 cells, while all screened cells displayed 7,9-O-Ac and 9-O-Ac Sias. Human cell lines appeared to predominantly display 7,9-O-Ac and 9-O-Ac Sias intracellularly, while MDCK cells displayed clearly on the membrane surface. Esterase treatment ablated probing for the modified Sia form (MDCK for 9-O-Ac inset box).

The 4-O-Ac Sias were found in cultured cells derived from horses (NBL-6), with between 10% and 30% of the cells showing positive staining results by microscopy (Fig. 3) and flow cytometry (data not shown). The 4-O-Ac Sias were not detected on the human (HEK-293, A549) or canine (MDCK) cells examined (Fig. 3).

The 9-O-Ac and 7,9-O-Ac Sias were widely but variably displayed on cells from different hosts, including several different human and animal cell lines (Fig. 3). The specificity of the SGRPs for their modified Sia target on cells was confirmed by 9-O-acetylesterase-active treatment of MDCK cells prior to PToV HE-Fc probing (Fig. 3, inset box). MDCK cells, which are frequently used for passage, amplification, and titration of influenza viruses, display both 7,9-O-Ac and 9-O-Ac Sias on their plasma membranes. Human cell lines (A549 and HEK-293) also synthesize 7,9-O-Ac and 9-O-Ac Sias, but the majority was detected at intracellular sites, likely within the Golgi/endoplasmic reticulum (ER) network based on their colocalization with GM130 as detected by antibody staining (data not shown). The intracellular localization of 9-O-Ac Sias in those human cells has been previously described (14). A horse cell line (NBL-6) displayed both 7,9- and 9-O-Ac Sias in addition to the 4-O-Ac Sias, and those were primarily detected on the cell surface.

### The distribution of Sias in mouse respiratory tissues.

**FIG 4:**
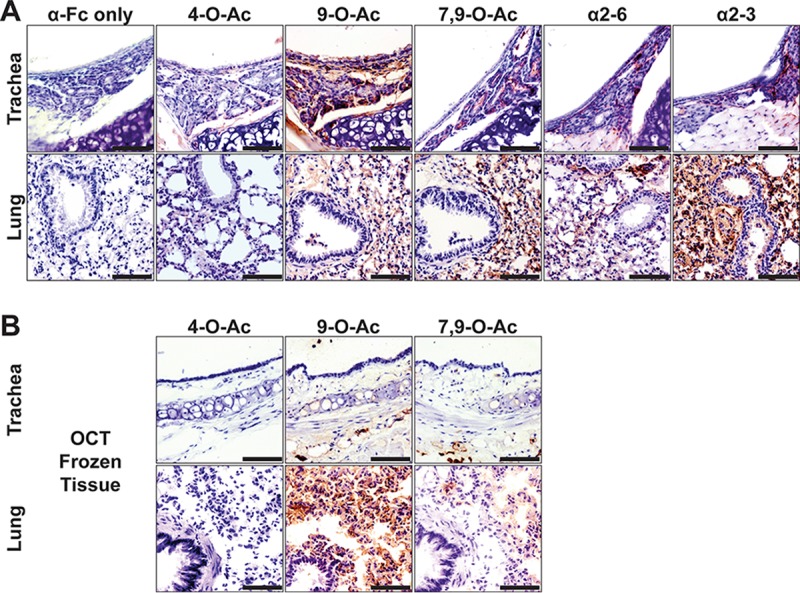
(A) Staining (red) of FFPE mouse respiratory tissues for the distribution of O-acetylated modified forms and Sia linkages. Controls with anti-human IgG Fc-specific antibody do not show background staining. Trachea and lung of mice were highly enriched in 9-O-Ac (and 7,9-O-Ac) Sias, including at the tracheal epithelium and lung pneumocytes. The α2-3-linked Sias are enriched in the mouse lung. (B) Staining (red) of frozen mouse respiratory tissues for O-acetylated modified Sias shows detection results similar to those seen with the FFPE tissue. Bars, 50 μm.

To generally confirm the binding of SGRPs and plant lectins to Sia forms in tissues, as well to determine their distribution in a key laboratory animal, we surveyed the respiratory tissues of C57BL/6 mice by histochemistry (Fig. 4). Mice showed little 4-O-Ac Sia in the respiratory tract tissues, with some signal seen in the lamina propria of the trachea and at pneumocytes in the lung, but high levels were displayed in the tissues of the small and large intestines (results not shown). Mice displayed 9-O-Ac (and 7,9-O-Ac) Sias in the trachea, often in the submucosal glands but also at some tracheal epithelium, and in the lung at pneumocytes of alveolar tissue. We also observed that the 9-O-Ac Sias were often distributed on endothelial linings of vessels and on erythrocytes preserved in the tissue sections. Lectin stains identified limited α2-6-linked Sias (SNA), with more-robust staining for α2-3-linked Sias (MAH) being particularly abundant in the lungs (Fig. 4A). The same general Sia identification patterns were observed in frozen tissue sections as in paraffin-embedded (PE) tissues. However, we observed staining variance in that frozen sections had both lower signal in the trachea and higher levels in the lung of 9-O-Ac Sias than in formalin-fixed, paraffin-embedded (FFPE) tissue and also showed some staining of the mucus that was still present on the slides (Fig. 4B).

### The distribution of 4-O-acetyl Sias in mammalian influenza virus host respiratory tissues.

**FIG 5:**
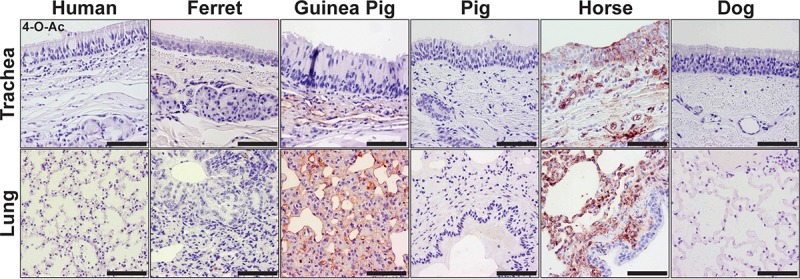
Staining (red) of mammalian respiratory tissues for the distribution of the 4-O-Ac Sia, detected with the MHV-S HE-Fc SGRP. Tracheal and lung sections were isolated from human, ferret, guinea pig, pig, horse, and dog. 4-O-Ac Sias were predominately found in the respiratory tissues of horses and guinea pigs, while trace signal was seen in ferret and dog tissues. Human and pig tissues appeared to contain no 4-O-Ac Sias. Bars, 50 μm.

The MHV-S HE-Fc SGRP showed a widespread display of 4-O-Ac Sias in the tracheal and lung tissues of horses and guinea pigs (Fig. 5). In horses, the 4-O-Ac Sia was displayed in the airway-exposed respiratory cells, including tracheal epithelia and alveolar pneumocytes, and that therefore would likely be in contact with infecting influenza viruses. Within the guinea pig respiratory tract, 4-O-Ac Sias were not seen at the tracheal epithelium but rather were displayed in the submucosa at the endothelial cells lining vessels. The lungs displayed significant 4-O-Ac Sia staining at alveolar pneumocytes (and endothelia, again). We observed display of 4-O-Ac Sias in small numbers of cells within ferret and dog tissues, but with little consistency or organization, and we did not observe any 4-O-Ac Sias in the examined respiratory tissues from humans or pigs.

### The distribution of 9- and 7,9-O-acetyl Sias in mammalian influenza virus host respiratory tissues.

**FIG 6:**
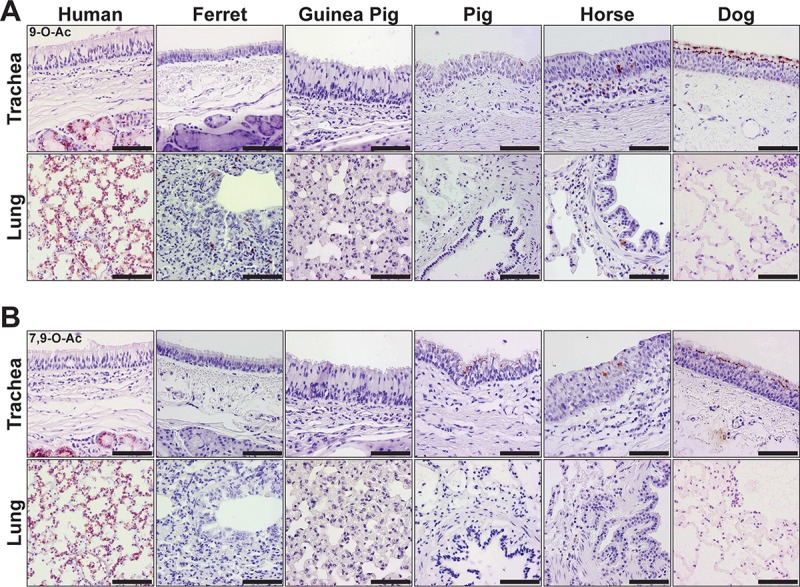
(A) Staining (red) of mammalian respiratory tissues for the distribution of the 9-O-Ac Sia, detected with the PToV-P4 HE-Fc SGRP. (B) Staining of respiratory tissues for the distribution of 7,9-O-Ac Sia, detected with the BCoV-Mebus HE-Fc SGRP. Both Sia forms were identified in respiratory tissues of all species examined, with variations in distribution and display. The 9-O-Ac and 7,9-O-Ac Sias generally appeared to have similar patterns of distribution. Human tissues displayed 7,9- and 9-O-Ac Sias in the tracheal submucosal glands and throughout the lung. These Sia forms were displayed at the tracheal epithelia of pigs, horses, and dogs. Bars, 50 μm.

We probed respiratory tissues using PToV-P4 HE-Fc to locate 9-O-Ac Sias (Fig. 6A) and BCoV-Mebus HE-Fc to locate 7,9-O-Ac Sias (Fig. 6B). The 7,9- and 9-O-Ac Sias were present in tissues of all mammals screened here, but with distinct tissue and cell display patterns. Human respiratory tissues displayed these modified Sias in both the trachea and lung. Within the human trachea, 7,9- and 9-O-Ac Sias were localized within submucosal glands, which are involved in excretion of respiratory mucins. Within the human lung, they were displayed on alveolar pneumocytes, as well as endothelia of vessels. The ferret lung displayed some 7,9- and 9-O-Ac Sias, while few to none were detected in the examined guinea pig respiratory tissues. In the respiratory tracts of pigs, horses, and dogs, 7,9- and 9-O-Ac Sias were displayed within the tracheal epithelial cells. Overall, 7,9- and 9-O-Ac Sias appeared most abundant within the respiratory tissues of humans in comparison to the other mammalian hosts surveyed.

### The distribution of Sia linkages in mammalian influenza virus host respiratory tissues.

**FIG 7:**
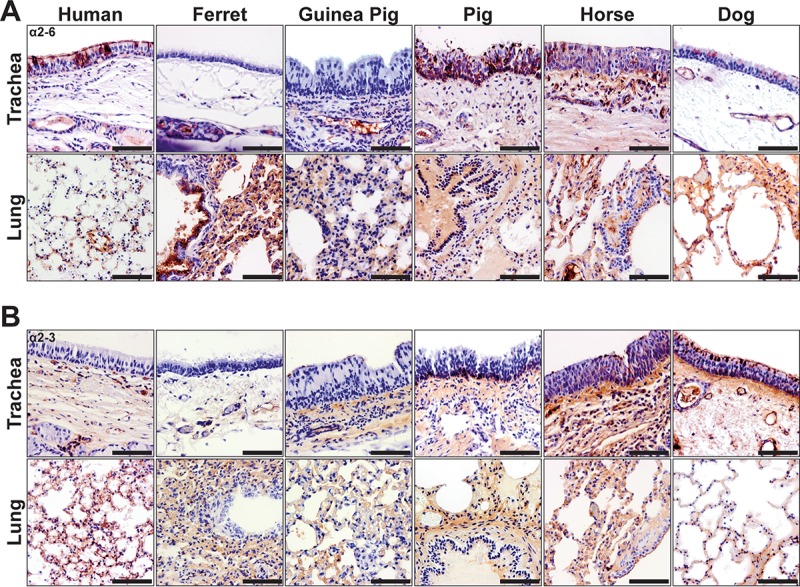
(A) Staining (red) of mammalian respiratory tissues by SNA for analysis of the distribution of α2-6-linked Sias. Human tissues show abundant staining at the tracheal epithelium, similarly to pig tissues. Ferret tissues display large amounts of α2-6-linked Sias in the submucosal glands and lung. (B) Staining (red) of mammalian respiratory tissues by MAH for analysis of the distribution of α2-3-linked Sias. These Sia linkages are displayed in the human lung alveolar tissue. They were found in all species examined but were particularly enriched in dog tissue such as the trachea. Bars, 50 μm.

Staining of mammalian respiratory tissues with plant lectins (α2-6-linked Sias [SNA], α2-3-linked Sias [MAH]) for Sia linkages confirmed a significant presence of Sias on the tissue sections (Fig. 7). Human respiratory tissues had abundant α2-6-linked Sias (SNA) at the tracheal epithelium (Fig. 7A), while an increasing level of the α2-3-linked forms (MAH) was seen in the lung (Fig. 7B). Ferrets displayed far more α2-6-linked Sias in the lung and in the submucosal glands of the trachea, while levels of α2-3-linked Sias appeared limited at vessels and in the lung alveolar tissue. Guinea pigs displayed both Sia linkages, though α2-3-linked Sias were more likely to be detected at the tracheal epithelium. Pigs displayed abundant α2-6-linked Sias at the tracheal epithelium, while both linkage forms were found throughout the tissues. Horses showed fairly equivalent levels of both Sia linkages in the respiratory tissues examined here. The tracheal epithelium of dogs was enriched for α2-3-linked Sias, where staining of cilia was easily observed.

### The distribution of O-acetyl Sias in Pekin duck respiratory and gastrointestinal tissues.

**FIG 8:**
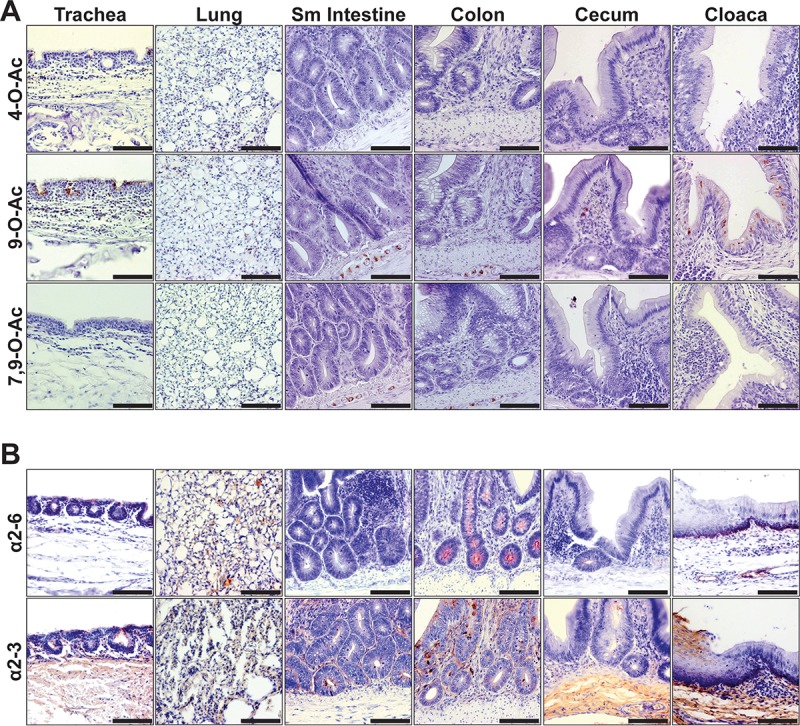
The distribution of Sias in the tissues of a Pekin duck, a major waterfowl reservoir of avian influenza viruses. (A) Staining (red) of respiratory tissues with SGRPs for O-acetyl modified Sias. Both 4-O-Ac and 9-O-Ac Sias are displayed at tracheal epithelia and in lung alveolar tissue. Within the digestive tissues, trace 7,9- and 9-O-Ac Sias are displayed in enteric ganglia of the intestines (Sm intestine, small intestine), whereas epithelial cells of the cloaca display abundant mono-9-O-Ac Sias. (B) Staining (red) of respiratory tissues for Sia linkage chemistry. Respiratory tissue displayed both Sia linkages, whereas digestive tissues showed more predominant α2-3-linked Sias. Bars, 50 μm.

Waterfowl of many types are the primary ecological hosts for influenza virus diversity in nature (59). Here we probed for O-acetylated Sias in respiratory (trachea, lung) and gastrointestinal (small intestine, colon, cecum, cloaca) tissues of Pekin duck (*Anas platyrhynchos domesticus*) and found the display of all O-acetyl Sias (Fig. 8A). By probing with MHV-S HE-Fc, we showed that 4-O-Ac Sias were displayed within a few epithelial cells of the trachea and alveolar pneumocytes of the lung but that none were observed within the digestive tract tissues examined. 9-O-Ac Sias were detected on the tracheal epithelia and within the lung pneumocytes in parallel to the 4-O-Ac, but we did not see a similar respiratory display of the di-7,9-O-Ac Sia form. Within the cloaca, we identified mono-9-O-Ac Sias within mucosal epithelia cell bodies lining the cloacal opening. Within the intestines and colon, enteric ganglia displayed 7,9- and 9-O-Ac Sia forms. An additional reported site of influenza replication, the pancreas, was screened with no display observed (data not shown). Examination of Sia linkage by lectin staining (SNA, MAH) confirmed the abundant presence of Sias in the tissue sections (Fig. 8B). Respiratory tissues tended to display both α2-3-linked and α2-6-linked Sias, while α2-3-linked Sia forms predominated in gastrointestinal tissues at the epithelia. One exception was the enriched display of α2-6-linked Sias within colonic crypts.

### The distribution of O-acetyl Sias in embryonated chicken eggs.

**FIG 9:**
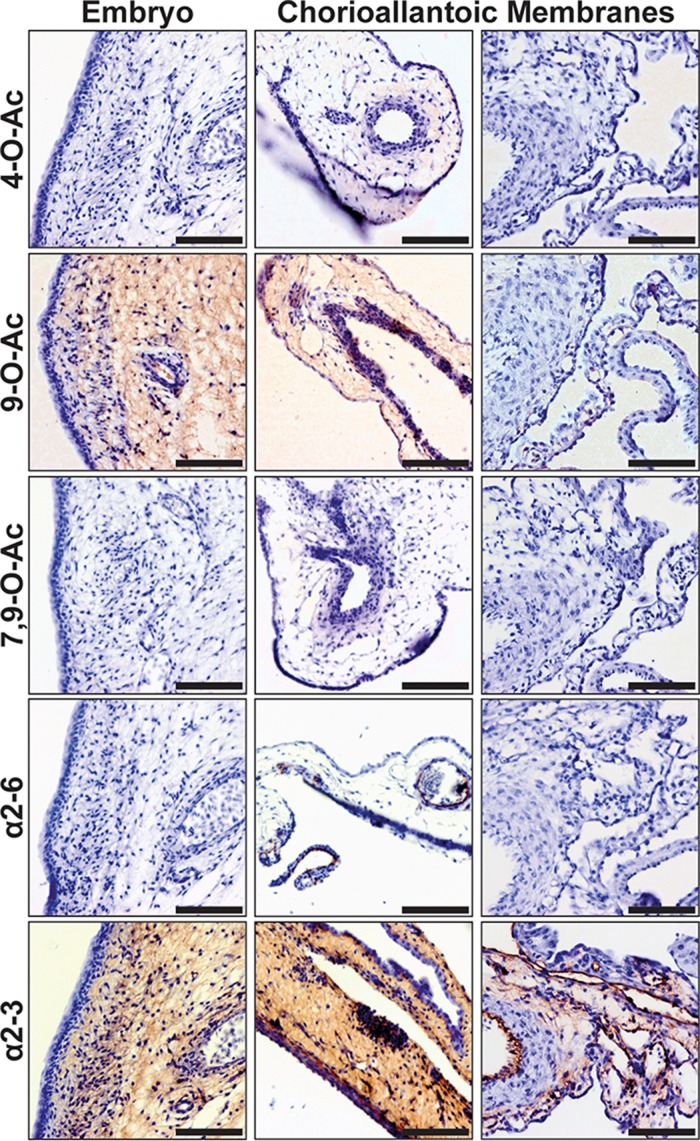
The distribution of Sias in tissues from 10-day postfertilization embryonated chicken eggs. Staining (red) of embryonic tissues and extraembryonic chorioallantoic membranes (CAM) is shown. Only mono-9-O-Ac Sias were detected in embryonic tissue and on CAM. There was significant staining for α2-3-linked Sias in the embryo and on membranes and limited presence of the α2-6-linked forms. Bars, 50 μm.

Embryonated chicken eggs are used for the amplification of many influenza viruses for diagnostic and experimental studies and for vaccine production, and they have been shown to display primarily α2-3-linked Sias, resulting in rapid selection of human strains (33, 60). We surveyed both embryonic tissue and a sampling of extraembryonic chorioallantoic membranes (CAM). Staining performed with PToV-P4 HE-Fc showed that mono-9-O-Ac Sia was present at high levels in the embryonic tissues and the extraembryonic membranes (Fig. 9). However, BCoV-Mebus HE-Fc failed to stain those tissues for the 7,9-O-Ac form, and the 4*-*O-Ac Sia form was also not observed. A solid-phase assay performed on allantoic fluid extracted from the eggs failed to identify any O-acetylated Sias (data not shown), suggesting that the 9-O-Ac Sias are primarily expressed on the cells of the embryos and the membranes. Staining with lectins for Sia linkage largely confirmed the significant presence of α2-3-linked Sias (MAH) in both the embryo and CAM, with only trace signals for α2-6-linked Sias (SNA).

## DISCUSSION

The overall goal of this study was to survey for the expression of the different modified forms of Sia on the cells and tissues of animals that are natural or experimental hosts of influenza viruses. We were able to confirm and extend prior work which showed that 4-O-Ac, 9-O-Ac, and 7,9-O-Ac Sias are all widely but very variably distributed in the target tissues of animals that are hosts for influenza virus, on the cells of chicken embryos, and on the cultured cells that are frequently used to propagate influenza viruses *in vitro*. In general, 7,9-O-Ac and 9-O-Ac Sias were broadly present across various species and their respective tissues and cell lines, while the 4-O-Ac Sias were widely detected in the examined tissues from some animals (horses and guinea pigs) but were not detected in the tissues from others (humans and pigs). We further established a direct comparison of the distributions of Sia linkages, α2-6 and α2-3, in these tissues using plant lectins SNA and MAH, respectively. Our findings in this study are summarized in Table 1.

**TABLE 1:** Summary of results of survey of influenza virus hosts for presence of varied Sia forms^a^

Host	4-O-Ac (MHV)	9-O-Ac (PToV)	7,9-O-Ac (BCoV)	α2-6 (SNA)	α2-3 (MAH)
Mouse, BL6	+	++	++	+	++
Human	−	++	+	++	+
Ferret	+/−	+	+	++	+
Guinea pig	++	+/−	+/−	+	+
Pig	−	++	+	++	+
Horse	+++	+	+	++	++
Dog	+/−	++	+	+	++
Duck, Pekin	+	++	+	+	++
Chicken, egg	−	++	−	+/−	+++

a +++ >>> +, detected (relative qualitative abundance and distribution); +/−, trace detection (no clear distribution or patterning); −, not detected.

The cell lines examined are commonly used for influenza virus laboratory isolation and growth studies, as are embryonated chicken eggs. Tissues sampled in the initial screening included those that are commonly infected by influenza viruses as well as by other respiratory viruses and bacteria. The respiratory tract tissues examined were those from natural or laboratory animal hosts of influenza viruses, while the intestines of ducks—which are among the natural reservoirs of those viruses—were also examined. The goal of this study was to form an initial picture of the distribution of the modified Sias, some of which have been reported to interfere with receptor binding and sialidase activity, to more clearly reveal their possible impact on viral replication and transmission. The results provide initial data that can be used for the design of studies for examining in detail the effects of the Sia modifications on influenza virus infection and spread.

### Display of 4-O-Ac Sias.

The first conclusion was that 4-O-Ac Sias are displayed in the respiratory tract tissues of guinea pigs and horses at high levels, as was likely based on previous reports of the presence of modified Sias on eyrthrocytes and blood proteins from those hosts (37, 61, 62). We observed some display of 4-O-Ac Sias in mice, but the amount seen in respiratory tissues was not as great. Previous reports (and our own unreported survey) showed that 4-O-Ac Sias in mice are highly tissue regulated and most abundantly found in the gastrointestinal tract (14). That Sia form was also identified in a very small number of cells within the duck, dog, and ferret respiratory tissues screened but not in the tissues of humans and pigs, and it appears not to be displayed on a variety of tissues examined in other studies in humans (14). We do not yet know the gene(s) encoding the 4-O-acetyltransferase, so it is unclear whether the trace display or absence of this modified Sia represents a genetic loss of the modifying enzyme in some species or variably regulated expression. The high levels of display of 4-O-Ac Sias in horses and guinea pigs are of interest, as horses have been the natural host of the H7N7 influenza virus, of two different H3N8 equine influenza viruses, and of an unknown influenza strain that spread widely around the year 1872 (63), while guinea pigs are naturally infected by and can transmit many strains of human influenza virus, including both H1N1 and H3N2 lineages (64). The presence of 4-O-Ac Sias in the respiratory tract therefore appears not to be a high barrier to at least some influenza virus infections, and it is currently not known whether their presence impacts the functions and evolution of HA and NA within the infecting influenza virus populations. The guinea pig tissues examined here did not display the 4-O-Ac Sia on airway-exposed tissues in the upper respiratory tract (trachea), so its role in laboratory infections performed with human viruses is unknown. Horse and guinea pig sera have long been known to be potent inhibitors of several H2 and H3 human influenza strains (35, 36, 65), and resistance to those sera could be selected through mutations in the HA at residue 145 (H3) that ablate 4-O-Ac binding (61). The presence of various amounts of sialidase-resistant Sias may therefore contribute to selection of influenza virus populations with HA variants, while its display on some horse and guinea pig cells in culture may provide an *in vitro* system suitable for the study of this modified Sia and its interactions with influenza and other viruses. Little is known about the direct effects of 4-O-Ac Sia on other microbes and pathogens, but their effects on sialidase (neuramindase) function would likely alter susceptibility in many respiratory viral and bacterial pathogens (66–68).

### Display of 9-O-Ac and 7,9-O-Ac Sias.

Both 9-O-Ac and 7,9-O-Ac Sias were widely displayed within the respiratory tissues of the influenza virus hosts, although with various locations and abundance levels. Previous studies performed using the influenza C HEF protein or HE-Fc proteins from bovine coronavirus and porcine *Torovirus* as probes for 7,9-O-Ac or 9-O-Ac Sias showed that they were widely displayed with significant variations (8, 14). The finding that high levels of both 9-O-Ac and 7,9-O-Ac Sias are found in the respiratory tracts of humans suggests that those may be a natural ligand for the human influenza viruses, as does the finding of both those modified Sias on MDCK cells and of the 9-O-Ac Sia in embyonated chicken chorioallantoic membrane (CAM) cells. The 9-O-Ac Sias are the primary receptor for influenza C viruses, so their expression in the respiratory tissues of humans and on chicken egg membranes allows infection by that virus (69). However, the 9-O-Ac group may block binding of some viruses, including strains of influenza A virus. The presence of 9-O-Ac Sias in the submucosal glands of the human trachea suggests that they would be displayed in mucus as part of the biophysical barrier to influenza virus contacting epithelial cell receptors (70–73). The animal with the distribution of 9-O-Ac Sias that most closely mirrored that of humans was the mouse. Their specific distribution in the respiratory tracts of other mammalian species was more varied but included display in cells of the tracheal epithelium of pigs, horses, and dogs. The influenza D virus group also utilizes the 9-O-Ac Sia receptor, indicating that this Sia form is present in functional amounts in the respiratory tracts of at least pigs, cows, and other ruminants (45), and 9-O-acetyl forms are present at high levels in bovine submaxillary mucin (Fig. 1). In contrast, we were unable to detect 9-O-Ac Sias in the given respiratory tissues (trachea and lung) of guinea pigs despite their ability to support influenza D virus infection (74). It is possible that this receptor form is more abundant in the far upper respiratory tract, such as in the nasal cavity. Display in duck tissues of both the respiratory tract and gastrointestinal tract showed that such Sias would represent a Sia form encountered in the avian influenza virus ecological life cycle. The recent identification of a 9-O-acetyltransferase (CAS1 domain-containing protein 1 [CASD1]) will allow a closer examination of regulated expression of the modifying enzyme and the impact on tissue-specific display of 7,9- and 9-O-Ac Sias (10). In addition, genetic manipulation of expression in cells will allow testing of the functional effects of the modification.

### Roles of modified Sia in controlling interactions with infectious pathogens.

Roles of modified Sia in altering the interactions of pathogens with hosts have been known or suggested for many decades—and some strong evidence of effects on influenza virus infection has been reported (31, 39, 61). Along with the well-known differences in the linkages of the Sias, the modified Sias may further impact influenza viruses at the population level, selecting for HA variants of Sia-binding avidity that may also have additional effects on antigenicity *in vivo* (75).

An additional common modified Sia is *N*-glycolylneuraminic acid (Neu5Gc), which is catalyzed from a Neu5Ac-CMP precursor by a CMP-N-acetylneuraminic acid hydroxylase (CMAH) (76). This modified Sia is present at high levels in many natural influenza virus hosts, including horses and pigs, as well as laboratory hosts such as mice and guinea pigs (77). However, CMAH has been independently lost by mutation in several influenza virus hosts, including humans and ferrets and likely in Western breeds of dogs (76, 78, 79). Neu5Gc may to be present but at low levels in tissues of avian lineages (80–82), but as a CMAH homolog has not been found in birds, this expression may be due to metabolic incorporation from dietary sources. Previous reports have shown that Neu5Gc Sia presented on human cells allows influenza virus binding but may interfere with productive virion entry and infection of some viruses (83).

In these studies, we examined the distribution of the O-acetylated Sias using the new tools that have become available and did not specifically investigate the presence of the nonmodified forms or of other multiply modified forms, if those were present. That would require either the use of SGRPs that recognize only the nonmodified forms or HPLC analysis of tissue lysates for the presence of modified Sias. Other probes currently under development include CD22-Fc, which shows reduced or blocked Sia binding when the 9-O-Ac modification is present (84, 85). Previous HPLC analysis has allowed quantification of the ratios of the modified to unmodified Sias. In guinea pig liver tissue, it was seen that Neu5Ac comprises 85% of Sias and that Neu5Gc and Neu4,5Ac_2_ modified forms represent 5% and 10% of the remaining Sias, respectively (24). This may represent an underestimation of the levels due to O-acetyl instability and release during sample manipulation. Quantification by HPLC is an important part of Sia analysis in clarifying the relative levels of abundance but fails to identify and visualize the specific location of display of the modified Sia forms. In our future work, we will seek to explicitly control the levels of the modified Sias by esterase treatments and by modifying enzyme activities so that we can test their role in the infection and release of influenza virus and other viruses.

### Summary.

The distribution of several types of modified Sias and their roles in altering the interactions of pathogens with their hosts are still poorly understood decades after the first reports of effects on virus infection and other functions. The use of new tools such as those deployed here and other novel biochemical, genetic, and experimental approaches will allow quantitative analysis of the expression of various Sia moieties and of their effects on pathogens and host responses.

## MATERIALS AND METHODS

### Animal tissue samples.

Animals tested in this study are listed in Table 1. Tissues collected at necropsy were fixed in 10% phosphate-buffered formalin and were then trimmed and blocked for paraffin embedding. Chicken eggs were harvested at 10 days postfertilization for embryonic tissue and chorioallantoic membranes (CAM). The mice were of the C57BL/6 strain, and tissues were either frozen in an optimum cutting temperature (OCT) matrix or fixed in formalin as described for other animal tissues. All postfixation processing was undertaken at the Animal Health Diagnostic Center at the Cornell University College of Veterinary Medicine. Anonymous human tissue sections were obtained from the University of Hong Kong Department of Pathology (per J. Nicholls) or United States Biomax, Inc. (Derwood, MD). Human tissue samples are exempt from Institutional Review Board (IRB) review as they originate from anonymous diagnostic specimens.

### Expression and isolation of HE-Fc viral SGRPs.

The sequences for expressing the ectodomains of the hemagglutinin-esterase (HE) of three nidoviruses (MHV-S, PToV-P4, and BCoV-Mebus) (14, 86) were synthesized by GenScript (Piscataway, NJ), with various degrees of insect codon optimization. To generate probe molecules, esterase domains were inactivated by changing the active site Ser residue to Ala by Q5 site-directed mutagenesis (New England Biolabs). The HE proteins were linked to the baculovirus gp64 signal sequence peptide at their N termini, and the C terminus was fused to a linker containing a thrombin cleavage sequence, the Fc domain of human IgG1, and a 6-His sequence. Constructs were cloned into pFastBac-1 (Life Technologies, Inc.) and were then used to generate recombinant bacmids in DH10Bac following the manufacturer's protocol. Recombinant baculoviruses were recovered by transfection of the bacmids into Sf-9 insect cells using Cellfectin II (Life Technologies, Inc.). Viruses were then used to infect a suspension of High Five cells and supernatants harvested 2 to 3 days postinfection. Proteins were purified from the infected cell culture supernatants by binding to a HiTrap protein G HP column (GE Healthcare Life Sciences, Piscataway, NJ) and eluted with 0.1 M citrate (pH 3.0; pH neutralization to pH 7.8 with 1 M Tris, pH 9.0) using an Äkta fast protein liquid chromatography (FPLC) system (GE Healthcare Life Sciences). The HE-Fc-containing fractions were dialyzed in phosphate-buffered saline (PBS) and concentrated using 30-kDa Amicon Ultra-15 filters (EMD Millipore). Purified protein was observed by SDS gel migration as well as anti-human IgG Fc probing by Western blotting. Migration was retarded by the presence of glycosylation and confirmed by the use of various glycosidases (EndoH, PNGase, and *O*-glycosidase; New England Biolabs). Protein concentrations were determined from *A*_280_ readings, and proteins were stored at −80°C in aliquots or at −20°C in 50% (vol/vol) glycerol.

### Solid-phase SGRP binding assay.

ELISA-grade 96-well plates were coated with Sia-containing samples diluted in PBS (pH 6.5; 100 μl/well) for 16 h at 4°C. Wells were washed between steps with washing buffer (PBS [pH 6.5], 0.05% Tween 20). Wells were blocked in CarboFree (Vector Laboratories) for 1 h at 37°C. HE-Fc SGRPs were precomplexed (10:1 molar ratio) with horseradish peroxidase (HRP)-conjugated goat anti-human Fc γ-specific antibody (Jackson ImmunoResearch, West Grove, PA) and were applied to wells in 2-fold serial dilutions (starting at 50 μg/μl HE-Fc) in blocking buffer (50 μl/well). Bound HE-Fc was detected with ABTS [2,2′-azinobis(3-ethylbenzthiazolinesulfonic acid)] substrate (Sigma). Optical densities were read at 405 nm. Samples depleted of specific O-Ac Sias were prepared by exposure to the respective active esterase HE-Fc forms at 20 μg/μl during blocking.

### Glycan microarray binding studies.

Glycan microarrays were fabricated using epoxide-derivatized slides as previously described (87). Printed glycan microarray slides were blocked by ethanolamine, washed, and dried. Slides were then fitted in a multiwell microarray hybridization cassette (AHC4X8S; ArrayIt, Sunnyvale, CA) to divide them into 8 subarrays. The subarrays were blocked with ovalbumin (1% [wt/vol])–PBS (pH 7.4) for 1 h at room temperature (RT), with gentle shaking. Subsequently, the blocking solution was removed and diluted protein samples with various concentrations were added to each subarray. After incubation of the samples for 2 h at RT with gentle shaking, the slides were washed. Diluted Cy3 AffiniPure goat anti-human IgG (H+L) antibody (purchased from Jackson Immuno Research Laboratories)-PBS was added to the subarrays, incubated for 1 h at RT, washed, and dried. The microarray slides were scanned by the use of a Genepix 4000B microarray scanner (Axon Instruments, Union City, CA). Data analysis was performed using Genepix Pro 7.0 analysis software (Axon Instruments, Union City, CA).

### Cell culture and staining of cultured cells.

All mammalian cells were grown at 37°C with 5% CO_2_. The cells tested included human HEK293T, human A549, canine MDCK, and equine NBL-6 cells. The cells were grown in Dulbecco’s modified Eagle medium (DMEM) supplemented with 10% fetal bovine serum (FBS). Insect cells (Sf-9 and High Five) were grown in Grace’s insect media supplemented with 10% FBS at 23°C. Cells were probed for O-acetyl Sias by the use of virolectin probes and visualized by indirect immunofluorescence. Cells grown on coverslips were fixed in 4% paraformaldehyde and blocked with CarboFree (Vector Laboratories). Esterase-active treatment controls were performed by addition of active HE-Fc (at 20 μg/ml) during blocking at 37°C. Cells were permeabilized with Triton X-100. Cells were probed with HE-Fc SGRPs (at 20 μg/ml) in complex with Alexa Fluor 488-conjugated goat anti-human Fc γ-specific antibody (10:1 molar ratio) for 45 min at RT. Colocalization to the secretory *cis*-Golgi network was determined by utilization of an anti-GM130 antibody (Sigma). Coverslips were mounted and visualized by fluorescence microscopy using a Nikon E3000 microscope.

### Histochemical staining of tissues.

Paraffin-embedded tissue sections on coated slides were dewaxed in xylene and rehydrated. Frozen sections of mouse tissues were placed directly on slides and then fixed with 10% buffered formalin. All subsequent washes were done with PBS–0.05% Tween 20 (PBS-T). Sections were first blocked in CarboFree (10% normal goat serum for SGRP probing) for 1 h at 37°C. Esterase treatment control slides were exposed to active esterase HE-Fc forms (20 μg/μl) during serum blocking. Sections were further blocked with avidin/biotin (Vector Laboratories, Burlingame, CA) and quenched of active peroxidase activity by the use of Bloxall (Vector Laboratories). Sections were probed with HE-Fc SGRPs (at 20 μg/ml) in complex with biotinylated goat anti-human Fc γ-specific antibody (10:1 molar ratio) overnight at 4°C. Sections were probed with biotinylated plant lectins SNA (Vector Laboratories) and MAH (MAA-II, Vector Laboratories) at 10 μg/ml and 20 μg/ml, respectively, for 3 h at RT. Sections were then exposed to ABC Vectastain strepavidin-HRP, followed by NovaRed HRP substrate (Vector Laboratories). Sections were counterstained in hematoxylin, dehydrated, and embedded in Cytoseal XYL.
